# Syringomyelia with Chiari I Malformation Presenting as Hip Charcot Arthropathy: A Case Report and Literature Review

**DOI:** 10.1155/2015/487931

**Published:** 2015-01-27

**Authors:** Roya Memarpour, Basheer Tashtoush, Lydia Issac, Fernando Gonzalez-Ibarra

**Affiliations:** ^1^Department of Pulmonary and Critical Care Medicine, Cleveland Clinic Florida, 2950 Cleveland Clinic Boulevard, Weston, FL 33331, USA; ^2^Ross University School of Medicine, 630 US Highway 1, North Brunswick, NJ 08902, USA; ^3^Department of Internal Medicine, Mount Sinai School of Medicine, Jersey City Medical Center, 355 Grand Street, Jersey City, NJ 07302, USA

## Abstract

Neuroarthropathy (neuropathic osteoarthropathy), also known as Charcot joint, is a condition characterized by a progressive articular surface destruction in the setting of impaired nociceptive and proprioceptive innervation of the involved joint. It is seen most commonly in the foot and ankle secondary to peripheral neuropathy associated with diabetes mellitus. Cases of hip (Charcot) neuroarthropathy are rare and almost exclusively reported in patients with neurosyphilis (tabes dorsalis). We report a case of a 36-year-old man who presented to the emergency department complaining of right hip pain. On physical examination, pain and thermal sensory deficits were noted in the upper torso with a cape-like distribution, as well as signs of an upper motor neuron lesion in the left upper and lower extremities. A magnetic resonance imaging study (MRI) of the right hip showed evidence of early articular surface destruction and periarticular edema consistent with hip Charcot arthropathy. An MRI of the spine revealed an Arnold-Chiari type I malformation with extensive syringohydromyelia of the cervical and thoracic spine.

## 1. Introduction

Neuroarthropathies reported in syringomyelia are rare and late complications of the disease, which often present as a monoarticular neuroarthropathy involving the upper extremities (shoulder and elbow joints) [1, 2]. We herein report a very rare presentation of undiagnosed syringomyelia with Chiari I malformation where hip Charcot arthropathy represented an early manifestation of the patient's underlying disease. In this paper, we discuss syringomyelia and neuropathic osteoarthropathies, with a brief review of published literature on their pathophysiology and association.

## 2. Case Presentation 

A 36-year-old Hispanic man presented to the emergency department complaining of right groin pain for one week. He described the pain as aching (6–8/10) in severity, localized to the right groin, precipitated by walking, and relieved by resting for approximately 10 minutes. The pain had progressively worsened over the previous week, and causing a painful limp at the time of presentation, he denied any morning joint stiffness, muscle aches, other joint pain, rash, fever, or back pain. He had taken a few tablets of nonsteroidal anti-inflammatory drugs every day for the last week, with minimal pain relief.

Upon review of other organ systems the patient described that he had been feeling subtle weakness in the left upper and lower extremities which started three years earlier and became slowly progressive. He stated that the weakness was not causing any limitation of activity or falls and denied any history of urinary or bowel incontinence, numbness, or pain in the extremities other than the presenting complaint.

When evaluated for the weakness two years earlier an MRI of the brain was ordered and reviewed by his primary care physician with reportedly normal results.

On physical examination, no erythema or swelling around the right hip or groin area, and no inguinal lymphadenopathy were noted. The passive range of motion of the right hip was limited and painful, especially forced internal rotation. Passive internal and external rotation of the extended leg “log roll test” and straight-leg raise test elicited pain. Bilateral hyperpigmentation from the knees to the toes was noted, with mild bilateral lower extremity pitting edema, and normal peripheral pulses.

Cranial nerves examination revealed a left spinal accessory nerve lesion as the patient was unable to shrug his left shoulder. Tongue fasciculation was noted with a slight deviation of the tongue to the left on protrusion, suggesting a left hypoglossal nerve lesion. The remainder of cranial nerves examination was normal with negative meningeal signs.

Motor examination revealed left upper and lower extremity weakness with a power of 4/5, in the right upper and lower extremities the power was normal 5/5. Deep tendon reflexes were exaggerated in the left upper and lower extremities and normal on the right. Increased muscle tone in the left upper and lower extremities was also noted, with a positive Babinski sign and ankle clonus on the left side. No muscle atrophy or other muscle fasciculations were identified.

Sensory examination revealed loss of vibration and position sensation in the left lower extremity, pain and temperature sensations were impaired in the left arm, and in a cape-like distribution across the shoulders and upper torso, anteriorly to the level of T2 dermatome and posteriorly to L1 dermatome. On rectal exam, the patient had a normal sphincter tone, with no evidence of prostate tenderness or other pathologies. Serological tests for syphilis, that included serum RPR and VDRL, were negative, with normal serum chemistry, B12, folate level, and complete blood count.

**Figure 1:**
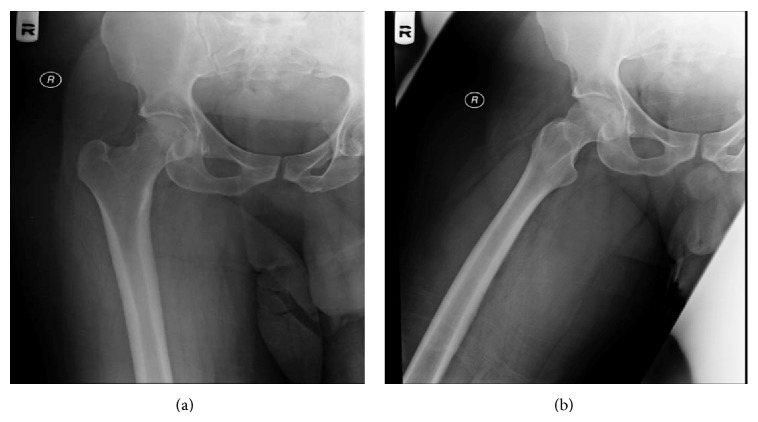
Right hip joint X-ray, (a) anteroposterior view and (b) abduction view.

Right hip X-ray (Figure 1) appeared to be normal with no fractures or dislocations and a preserved articular space.

**Figure 2:**
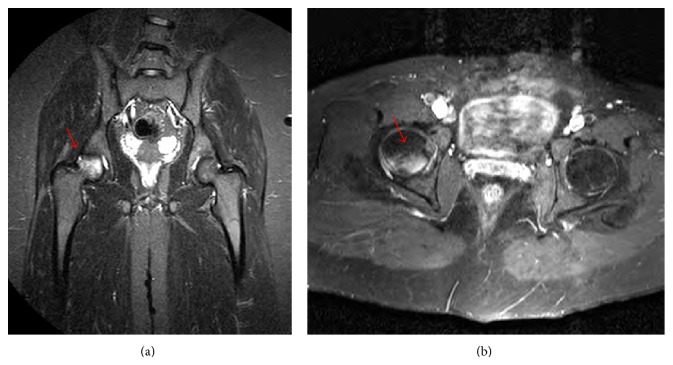
Hip MRI (a) coronal view and (b) axial view. A subcortical wedge shaped enhancing area (*arrows*) with adjacent bone marrow edema in the right femoral head posterior articular surface.

Right hip joint MRI (Figure 2) showed a wedge shaped subcortical enhancement with adjacent bone marrow edema in the right femoral head posterior articular surface, suggestive of avascular necrosis or early radiological signs Charcot arthropathy considering the patients underlying neurological disease.

MRI of the brain was normal, with no evidence of infarcts, atrophy, or any other intracranial pathology.

**Figure 3:**
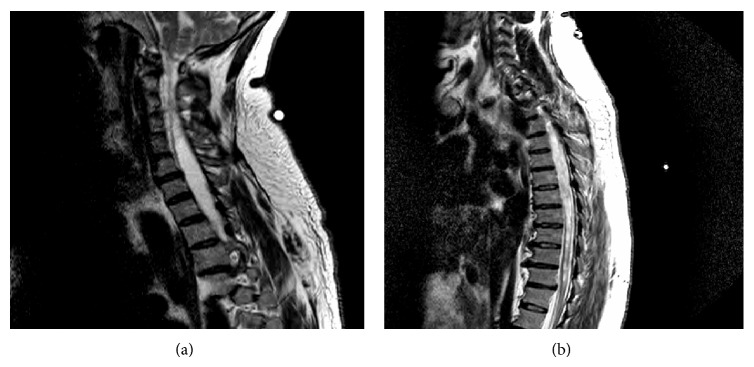
(a) MRI of the cervical spine showing a 9 mm inferior herniation of the cerebellar tonsils through the foramen magnum (Type I Chiari Malformation), with extensive cervical and superior thoracic spinal cord syringohydromyelia. (b) Thoracic spine MRI revealing extensive syringohydromyelia involving the entire thoracic spinal cord. The syringohydromyelia is more prominent in the superior midportion of thoracic spinal cord. T11-T12 central disk protrusion with thecal sac compression and slight ventral spinal cord compression.

MRI of the cervical and thoracic spine (Figure 3) showed a 9 mm inferior herniation of the cerebellar tonsils through the foramen magnum, compressing the craniocervical junction (Type I Chiari Malformation) and extensive cervical and thoracic spinal cord syringohydromyelia.

The patient underwent neurosurgical intervention, where cervical decompression with a suboccipital craniectomy and C1 laminectomy was performed. He was discharged to a rehab facility where regular physical therapy and follow-up in an out-patient clinic was arranged. Over the following two years the patient was able ambulate with assistance due to slight progression of left lower extremity weakness (opposite to the site of the hip neuroarthropathy); he had no recurrent right hip pain or joint deformities, and no orthopedic surgery was required.

## 3. Discussion 

### 3.1. Syringomyelia

#### 3.1.1. Background and Classification

Syringomyelia is the development of an abnormal fluid-filled cavity “syrinx” within the spinal cord. Hydromyelia is dilatation of the central canal by an accumulation of cerebrospinal fluid (CSF). These two findings usually occur simultaneously and are referred to as syringohydromyelia; however, hydromyelia is often implied in the term syringomyelia. The prevalence is estimated to be 8.4 cases per 100,000 people and usually appears in the third or fourth decade of life, with a mean age of onset of 30 years [3].

Syringomyelia can be generally classified into four groups: communicating, noncommunicating, atrophic cavitation (syringomyelia ex vacuo), and neoplastic cavitation.

Communicating syringomyelia is caused by obstruction of CSF pathways, located distal to the fourth ventricular outlets, creating a syrinx that communicates with the fourth ventricle. In noncommunicating syringomyelia, the syrinx and dilated central canal do not communicate with the fourth ventricle, and the obstruction is usually located at or below the level of the foramen magnum [4]. The latter is the most common type of syringomyelia, found in approximately 50% of all cases, where CSF flow from the posterior fossa to the caudal space is blocked, and the most common underlying etiology is an Arnold-Chiari type I malformation [1, 5]. Other common causes of syringomyelia include hindbrain anomalies, neoplastic disease, inflammatory conditions, and trauma [6, 7].

#### 3.1.2. Pathophysiology

Although many mechanisms for syrinx formation have been postulated, the exact pathogenesis remains unclear. Frequently cited theories are those of Gardner, Williams, and Oldfield et al. [8–10].

Gardner's hydrodynamic theory proposes that syringomyelia results from a “water hammer-like” transmission of pulsatile pressure to the CSF during systole, forcing fluid out of the ventricles, via a communication between the fourth ventricle and the central canal of the spinal cord [8, 11].

Williams's theory proposes that syrinx development, particularly in patients with a Chiari malformation, follows a differential between intracranial pressure and spinal pressure caused by a valve-like or sucking action at the foramen magnum. The increase in subarachnoid fluid pressure from increased venous pressure during coughing or valsalva maneuvers is localized to the intracranial compartment, creating a craniospinal pressure gradient that draws CSF caudally into the syrinx [9, 12].

Oldfield's theory suggests that a downward movement of the cerebellar tonsils during systole, which can now be visualized with dynamic MRI, creates oscillations with a piston effect in the spinal subarachnoid space that acts on the surface of the spinal cord and forces CSF through the perivascular and interstitial spaces, resulting in edema of the spinal cord, and eventually creating a syrinx, that enlarges over time [10, 13–15]. Experimental findings by Stoodley et al. suggest that there is a preferential flow of CSF from the spinal cord perivascular spaces into the central canal by an arterial pulsation-driven mechanism in non-communicating syringomyelia [16].

#### 3.1.3. Presentation

The clinical presentation of syringomyelia is dependent upon both the location and the diameter of the syrinx within the spinal cord.

The classic presentation of syringomyelia is a shawl-like (cape-like) distribution of impaired pain and temperature sensation across the shoulders and upper torso.

The syrinx first interrupts the decussating spinothalamic fibers due to their lateral location and close proximity to the central canal, resulting in early loss of pain and temperature sensation, while light touch, vibration, and position senses are preserved. This is often referred to as “dissociated sensory loss.” An enlarging syrinx can also produce pain due to impingement of the spinothalamic fibers, which is commonly described as “dysesthetic pain,” which is a deep aching pain usually involving the neck and shoulders but may also follow a radicular distribution in the arms or trunk [17, 18].

As the cavity enlarges, it can involve the posterior columns, causing loss of vibration sense and proprioception in the lower extremities. Later in the disease course, a syrinx extension into the anterior horns of the spinal cord damages the lower motor neurons, causing diffuse muscle atrophy and flaccid paralysis that begins in the hands and progresses proximally to include the forearms and shoulder girdles [18, 19].

The syrinx may extend into the medulla, producing a syringobulbia or beyond the medulla into the brain stem and the centrum semiovale (syringocephalus). Horner syndrome may appear, reflecting damage to the sympathetic neurons in the intermediolateral cell column.

Lumbar syringomyelia is characterized by atrophy of the proximal and distal leg muscles with dissociated sensory loss in the lumbar and sacral dermatomes. Lower limb reflexes are reduced or absent. Impaired bowel and bladder functions usually occur as a late manifestation, and sexual dysfunction may develop in long-standing cases [18–20].

Other rare manifestations of syringomyelia include scoliosis [21, 22], painless ulcers of the hands, edema, and hyperhidrosis, due to the interruption of central autonomic pathways [23].

Neuropathic osteoarthropathy (Charcot joint) caused by syringomyelia is rare and a commonly misdiagnosed disease. Of the few cases reported in literature, the upper extremity (wrist, elbow, and shoulder) joints remain the most common site of syringomyelia related arthropathies [1, 2, 24–26].

In a recent study by Deng et al., clinical and imaging data of 12 patients with neuropathic arthropathy caused by syringomyelia over a 9-year period were reviewed. The study showed that 11 patients had an underlying Chiari malformation, sixteen joints were involved, including 10 elbows, 3 shoulders, 2 interphalangeal joints, and 1 wrist. The side of the syrinx on cervical axial MRI was consistent with the side of the affected limb. All patients who underwent neurosurgical treatment stated improvement in neurological dysfunctions and no deterioration in symptoms related to neuropathic arthropathy. No surgical management was performed on the neuropathic joints in any of their patients [1].

### 3.2. Charcot Joint (Neuropathic Osteoarthropathy)

In 1868, Jean-Martin Charcot gave the first description of the neuropathic aspect of this disease. Diabetes is considered to be the most common etiology of Charcot arthropathy. However, any condition that causes sensory or autonomic neuropathy can lead to a Charcot joint.

#### 3.2.1. Pathophysiology

Two main theories predominate in the pathophysiology of neuropathic osteoarthropathy. The neurotraumatic theory states that the arthropathy is caused by an unperceived trauma or injury to an insensitive functional joint. The second theory is the neurovascular theory, which suggests that the development of autonomic neuropathy causes an increased blood flow to the joint; this in turn results in a mismatch in bone destruction and regeneration, leading to joint hypertrophy and architectural distortion [27]. It is likely that a combination of these two processes plays a role in the development of a Charcot joint.

#### 3.2.2. Association and Distribution

In the upper extremity, syringomyelia, trauma, and neoplastic conditions tend to be the most common causes of the osteoarthropathy. In the lower extremity, diabetes, tertiary syphilis, alcoholism, and congenital insensitivity to pain are among the most common causes [28]. Diabetes has been found to be the most common cause of Charcot affecting the ankle and foot joints, with a prevalence of neuroarthropathy ranging from 0.08% in the general diabetic population to 13% in high-risk diabetic patients [29].

Charcot hip is a very rare and unique presentation of syringomyelia secondary to Chiari I malformation.

Charcot arthropathy involving the hip joint is generally uncommon. The majority of cases have been reported in association with tabes dorsalis, a late manifestation of tertiary neurosyphilis, where the incidence of neuropathic arthropathy in these patients ranges from 6% to 10% [30, 31]. A few cases of hip and knee joint neuroarhtropathies have been reported as rare complications of epidural anesthesia that caused lumbar syringomyelia [32, 33]. Other reports of hip Charcot arthropathies have also been described in patients with spina bifida [34], congenital insensitivity to pain [35, 36], and chronic alcoholism [37].

Unlike upper extremity syringomyelia induced neuroarthropathy, which typically involves the side of greater syrinx extension and motor weakness, Charcot hip opposite to the side of the weakness in our patient can perhaps be explained by the increased stress and abnormal weight bearing on the right hip joint while the patient was accommodating for his left lower limb weakness.

## 4. Conclusion

A detailed medical history and a careful physical examination are crucial elements in evaluating all patients with features of myelopathy regardless of the presenting complaint. Differentiating neuropathic arthropathy from other more common joint diseases, even though challenging, remains an essential step to prevent the progression of joint destruction, as the treatment profoundly relies on the early diagnosis and management of the underlying neurological disease.
